# Long‐Term Use of Anti‐Pronation Insoles Enhances Inter‐Joint Coordination in Individuals With Flat Feet

**DOI:** 10.1002/jfa2.70124

**Published:** 2026-01-09

**Authors:** Negin Soltani, Mahdi Majlesi, Ali Fatahi

**Affiliations:** ^1^ Department of Physical Education and Sport Sciences, Central Tehran Branch Islamic Azad University Tehran Iran; ^2^ Department of Sport Biomechanics Hamedan Branch Islamic Azad University Hamedan Iran

**Keywords:** anti‐pronation insole, flatfoot, gait, inter‐joint coordination, vector coding

## Abstract

**Background:**

Flatfoot alters lower limb biomechanics and can negatively affect interjoint coordination during gait. Antipronation insoles are commonly prescribed to correct excessive foot pronation, yet their long‐term effects on interjoint coordination remain unclear. This study aimed to examine whether prolonged use of antipronation insoles improves interjoint coordination in individuals with flat feet.

**Methods:**

Twenty‐four participants (12 with flat feet and 12 with normal arches) were included. Spatiotemporal and interjoint coordination parameters were evaluated under four gait conditions: normal gait (NG), posttest normal gait (PNG), walking with shoes (SH), and posttest walking with shoes (PSH). Gait kinematics and kinetics were recorded using a motion capture system and force plates. All participants completed a baseline gait assessment, after which the flat foot group (FFG) underwent a 6‐week intervention with antipronation insoles. A follow‐up gait assessment was conducted for both groups to determine the long‐term effects of insole use. Interjoint coordination was analyzed using the vector coding technique.

**Results:**

Following 6 weeks of insole use, the flat‐foot group showed phase‐dependent changes in ankle–hip coordination, with lower coupling angles than controls during loading response and swing and higher angles during push‐off under the normal‐gait condition (*p* ≤ 0.01 and *η* ^2^ *p* = 0.28–0.33). Changes in ankle–knee and knee–hip coordination were smaller and generally limited to specific gait phases. Insoles produced an overall increase in coupling angles across conditions, reflecting these phase‐specific effects. Spatiotemporal analysis showed longer single‐support times in controls, longer double‐support times in the flat‐foot group with insoles, greater baseline stride length in controls, and no between‐group difference in walking speed.

**Conclusion:**

Long‐term use of antipronation insoles improves interjoint coordination in distal lower limb joints and may contribute to a more conservative or steadier gait pattern in individuals with flat feet as reflected by phase‐specific changes in spatiotemporal parameters. However, their limited influence on proximal joints underscores the need for complementary interventions, such as targeted rehabilitation exercises.

## 1 Introduction

The foot is a complex structure that not only provides a stable support surface for standing and walking but also endures significant mechanical loads [1]. Given the interconnected nature of the musculoskeletal system, any alteration in one segment can affect other regions, potentially leading to secondary disorders [2]. Foot abnormalities are among the common contributors to pain, fatigue, and dysfunction in the lower limbs [3]. Flatfoot, one of the most prevalent foot deformities, is characterized by a reduced medial longitudinal arch and may result from the shortening of the peroneus longus muscle and elongation of the anterior and posterior tibialis muscles [4]. The arches of the foot play a crucial role in attenuating ground reaction forces, preventing their full transmission to the body. However, in individuals with flatfoot, the absence of a functional arch results in greater force transmission to the proximal joints, which, over time, may contribute to a cascade of musculoskeletal complications, particularly in the spine [5]. Flatfoot alters the kinematic behavior of the foot–ankle complex primarily through excessive subtalar pronation, which increases tibial internal rotation and modifies distal coupling patterns during stance. These distal deviations can propagate proximally through the kinetic chain, contributing to increased knee valgus, altered hip rotation strategies, and compensatory trunk adjustments during walking [6, 7, 8]. Such multilevel adaptations represent a coordinated response to chronic medial arch collapse rather than isolated segmental changes. Given these interconnected biomechanical effects, evaluating interjoint coordination provides a more comprehensive understanding of gait alterations in flatfoot. Unlike conventional kinematic or kinetic measures that assess joints independently, coordination analysis quantifies how ankle, knee, and hip joints interact throughout the gait cycle, revealing altered movement organization and compensatory coupling strategies that may not be detectable through isolated joint angles [9, 10]. Therefore, this approach offers unique insight into kinetic‐chain integration and the potential modulatory effects of antipronation insoles.

Gait symmetry is another important domain in the functional assessment of individuals with flatfoot. Altered foot structure and excessive pronation can disrupt interlimb balance and lead to asymmetrical loading patterns, resulting in compensatory adaptations throughout the lower kinetic chain. Recent studies have demonstrated that individuals with pronated or flatfoot postures exhibit increased asymmetry in joint kinematics, ground reaction forces, and spatiotemporal parameters, which may reflect inefficiencies in neuromuscular control [11, 12]. Although the present study focuses primarily on interjoint coordination, coordination analysis complements symmetry assessments by quantifying how joints interact across the gait cycle, thereby offering additional insight into movement organization that may not be captured solely through symmetry‐based metrics. Chen et al. (2010) reported that individuals with flatfoot exhibit increased rearfoot eversion and pronation, greater ankle plantarflexion, and enhanced knee flexion during walking [13]. Similarly, Eslami et al. (2009) found that excessive foot pronation during gait leads to increased internal tibial rotation, a decrease in medial longitudinal arch height, increased tibial abduction relative to the thigh, knee valgus positioning, and ultimately greater stress on knee ligaments and increased loading on lower limb joints [14]. Flatfoot‐related alterations are strongly mediated by abnormal motion of the subtalar joint, which acts as the primary controller of rearfoot eversion and the coupling between the foot and tibia during stance. Excessive subtalar pronation has been shown to increase tibial internal rotation, modify ankle dorsiflexion patterns, and contribute to compensatory knee valgus and altered hip mechanics throughout the gait cycle [15, 16]. These distal alterations can propagate proximally through the kinetic chain and may influence interjoint coordination strategies in individuals with flatfoot. Although the present study focuses on sagittal‐plane coordination between the ankle, knee, and hip, acknowledging the contribution of subtalar mechanics provides a more comprehensive biomechanical framework for interpreting coupling changes.

Among the various interventions for correcting flatfoot, antipronation insoles have been shown to partially improve joint function [17]. Among commonly used therapeutic approaches, orthopedic specialists recommend externally wedged (antipronation) insoles to help control excessive foot pronation [18, 19]. Research indicates that wearing antipronation insoles can immediately reduce ankle plantarflexion, decrease knee flexion, and increase foot supination. However, the precise corrective mechanisms of these insoles remain unclear. Furthermore, some studies suggest that their ability to reduce excessive pronation is minimal, with reported reductions of approximately 2° [20]. Orthotic insoles are widely used in the treatment of various lower limb injuries related to biomechanical inefficiencies [21]. They are designed to realign skeletal structures, modify lower limb movement patterns, and alleviate symptoms associated with foot abnormalities [22]. The immediate effects of orthotic insoles on biomechanical variables, such as kinematics and muscle activity, particularly in individuals with different anatomical foot structures, have become a key focus of research.

Exploring lower limb interjoint coordination during walking with and without orthotic insoles may provide valuable insights into their effectiveness [23]. Heiderscheit et al. (2002) introduced the vector coding technique as a method for continuously calculating joint coupling angles [24]. This approach assesses the relative motion between all data points using an angle‐angle plot, determining the coupling angle relative to the horizontal axis at each point. This process is repeated throughout the entire stance phase. Assessing interjoint coordination, particularly ankle–hip, knee–ankle, and knee–hip coupling, and the effects of antipronation insoles on these dynamics is of significant clinical importance. Lower limb interjoint coordination, especially between the ankle and hip, plays a crucial role in controlling foot positioning and maintaining the body's center of mass during walking. Variability in coordination during gait is linked to the health of biological systems [25]. Changes in interjoint coordination may indicate shifts in movement strategies, and evidence suggests that increased variability can signify a transition from one stable coordination pattern to another [26, 27]. This study aims to investigate the long‐term effects of antipronation insoles on interjoint coordination in individuals with flatfoot and compare them to those with normal foot arches. Based on prior studies linking excessive subtalar pronation to altered distal coupling and reduced push‐off efficiency, we hypothesized that long‐term use of anti‐pronation insoles would produce phase‐specific improvements in interjoint coordination. We expected reduced ankle–hip coupling during loading response and swing, reflecting attenuation of pronation‐related distal kinematic deviations, and increased ankle–hip coupling during push‐off, consistent with improved distal joint integration during propulsion. In contrast, only minimal or nonsignificant changes were anticipated in ankle–knee and knee–hip coordination as proximal joints are less directly influenced by medial arch support. This phase‐ and joint‐specific hypothesis aligns with the mechanistic influence of foot orthoses on distal joint control during gait.

## 2 Methods

### 2.1 Participants

A priori sample size estimation was performed using G*Power 3.1 [28]. An effect size of 0.40 was selected based on previous studies reporting medium‐to‐large effects in lower‐limb coordination and coupling‐angle variability among individuals with pronated or flat feet using vector coding approaches [26, 29]. The power analysis was conducted for a three‐way mixed repeated‐measures ANOVA design, incorporating one between‐subject factor (group: flatfoot vs. control) and two within‐subject factors (time: pre vs. post and walking condition: barefoot vs. shod), with *α* = 0.05 and statistical power set to 0.80. This analysis indicated a minimum required sample size of 24 participants. Twelve individuals with flat feet were subsequently recruited from orthopedic clinics at Bessat Hospital, Hamedan, forming the experimental group (FFG), and 12 age‐, height‐, and weight‐matched individuals with normal foot arches were selected as the control group (CG). Participants in the flat‐foot group were required to exhibit a navicular drop greater than 10 mm based on the procedure described by Lange et al. [30]. According to established clinical guidelines, an ND > 10 mm is considered diagnostic for flexible flatfoot as it reflects medial arch mobility between non–weight‐bearing and weight‐bearing conditions; this criterion does not apply to rigid flatfoot, which lacks measurable arch deformation [30, 31]. Therefore, all individuals meeting this threshold were classified as having flexible flatfoot. Exclusion criteria included a history of lower‐limb or trunk injury, prior surgery, fractures, burns, neuromuscular disorders, prosthetic limb use, diabetes, peripheral neuropathy, or previous use of insoles or orthopedic footwear [30] as well as any clinical indication of rigid flatfoot. To minimize the influence of muscular strength or training status on gait kinematics, only individuals with a low‐to‐moderate habitual physical activity level were included. Physical activity level was screened through a short prestudy interview in which participants confirmed the absence of structured athletic training (i.e., > 3 sessions per week) and engagement exclusively in routine daily activities or light recreational exercise, which is consistent with prior gait‐related research aiming to control for activity‐related confounding [32, 33]. All participants provided written informed consent before participation. The study was approved by the Research Ethics Committee of Islamic Azad University, Hamedan Branch (Approval Code: IR.IAU.H.REC.1402.130 and Approval Date: 19 November 2023) and was conducted in accordance with the 1964 Declaration of Helsinki and its subsequent amendments.

### 2.2 Instruments and Examination

In each subject, the following anthropometric data were measured: weight, height, leg length, and knee and ankle widths for both the left and right sides. These measurements were essential for input into the VICON Nexus 1.8.2 software to calculate the selected biomechanical parameters. Movement analysis was conducted using a Vicon motion capture system (Vicon, Oxford, UK) equipped with six T‐series cameras, capturing data at a sampling frequency of 100 Hz. Two Kistler force plates integrated into the floor (400 × 600 mm, Type 9281, Kistler Instrument AG, Winterthur, Switzerland) measured the ground reaction forces with a frequency of 1000 Hz. Spherical markers (diameter: 14 mm) were attached on the specific anatomical landmarks on both lower limbs with double‐sided adhesive tape. The marker placement followed the Plug‐In Gait model [23]. The cameras and force plates were calibrated within a predefined 3D space located at the center of a 12‐m walkway. Participants walked along the walkway at their self‐selected speed. To ensure natural gait patterns and minimize start and stop effects, the starting position was adjusted for each participant so that they took at least seven steps before entering and after exiting the calibrated zone. Prior to data collection, participants completed a 10‐min familiarization session to ensure natural gait patterns. During testing, each participant performed six trials, from which three successful trials were selected for further analysis based on consistent foot placement on the force plates.

**FIGURE 1:**
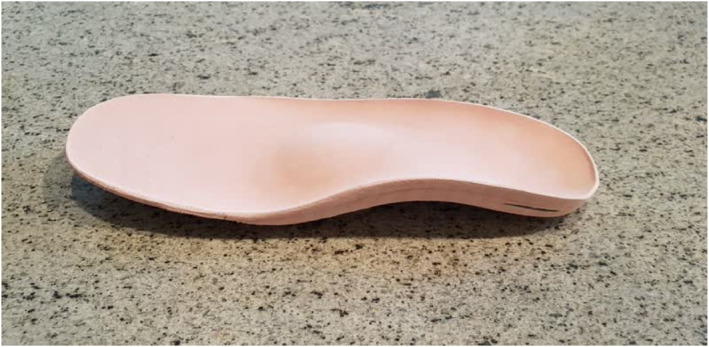
Structure and design features of the antipronation insoles used in this study (Arc Support FO, Longxin Industrial Ltd, LX‐0701‐1). The insoles are made of polyurethane material and include a medial wedge and firm arch support designed to reduce excessive foot pronation.

The experimental protocol included two walking conditions for all participants: barefoot and walking with shoes. In both groups, the shod condition was performed using the same standardized neutral‐cushioned athletic footwear (Adidas Duramo SL, Adidas AG, Herzogenaurach, Germany) to ensure consistency in shoe‐related mechanical characteristics. In the FFG, these shoes were fitted with antipronation insoles, whereas in the CG, they were used without insoles. Accordingly, four gait conditions were defined: normal gait (NG; barefoot, pretest), posttest normal gait (PNG; barefoot, posttest), walking with shoes (SH; shod, pretest), and posttest walking with shoes (PSH; shod, posttest). The antipronation insoles used in this study were prefabricated polyurethane insoles (Arc Support FO, Longxin Industrial Ltd., Model LX‐0701‐1) (Figure 1). These insoles featured a firm but flexible arch support and an integrated medial wedge of approximately 5° designed to control excessive pronation and promote optimal alignment of the medial longitudinal arch during gait.

To evaluate the long‐term effects of insole use, participants in the FFG were instructed to wear the insoles for a minimum of 6 h per day over a 6‐week intervention period during routine daily activities, such as walking, light ambulation, school or work tasks, and general household mobility. They were advised not to use the insoles during high‐intensity sports to minimize uncontrolled load variability. Compliance with the prescribed wearing schedule was monitored weekly through structured phone follow‐ups in which participants reported their approximate daily duration of insole use. Prior to the intervention, an experienced physiotherapist individually fitted each insole to ensure proper placement within the standardized footwear. All participants were instructed to use the same model of athletic shoes throughout both laboratory assessments and the intervention period to maintain consistency in footwear‐related mechanical influences.

After the intervention period, a posttest gait assessment was conducted to evaluate the effects of prolonged antipronation insole use. Kinematic data were filtered using a zero‐lag fourth‐order low‐pass Butterworth filter with a 6 Hz cutoff. Distinct gait events were identified in the sagittal plane to segment the gait cycle into the following phases: loading response (LR, 0%–15%), mid‐stance (MS, 15%–45%), push‐off (PO, 45%–60%), and swing (S, 60%–100%) [34]. All participants were right‐leg dominant, and no significant differences were observed between limbs during the preliminary analysis; therefore, all analyses were performed on the right leg. Data for each stride were time‐normalized to 100 points using linear interpolation. Interjoint coordination was quantified using the vector coding technique. Time‐normalized sagittal plane joint angles of the ankle, knee, and hip were used to construct angle–angle plots for each joint pair, and coupling angles were derived from the orientation of the vector connecting successive points on these plots following established procedures [24, 35]. Coupling angles (0°–360°) were subsequently classified into four coordination categories using standard angular thresholds: distal phase (DP: 0°–22.5°, 157.5°–180°, and 337.5°–360°), in‐phase (IP: 22.5°–67.5° and 202.5°–247.5°), proximal phase (PP: 67.5°–112.5° and 247.5°–292.5°), and antiphase (AP: 112.5°–157.5° and 292.5°–337.5°). Both coordination magnitude and coordination variability were analyzed. Coordination magnitude was defined as the mean coupling angle within each gait phase, whereas coordination variability was calculated using the coefficient of variation (CV) of coupling angles across the three valid trials for each participant, consistent with the method applied in previous work [27]. This approach captures trial‐to‐trial consistency in interjoint coordination and has been used in earlier gait coordination research [36, 37].

### 2.3 Statistical Analyses

All statistical analyses were performed using SPSS version 26.0 (IBM Corp., Armonk, NY, USA), with the significance level set at *p* ≤ 0.05. Data normality was verified using the Shapiro–Wilk test, and all variables met the normality assumption. A mixed‐model repeated‐measures ANOVA was used to examine the effects of time (pretest vs. posttest) and gait condition (NG vs. SH) as within‐subject factors, and group (flatfoot vs. control) as a between‐subject factor. This analysis allowed us to evaluate time × condition × group interactions while accounting for group‐level differences in foot posture. When significant main effects or interactions were detected, Bonferroni‐adjusted post hoc tests were applied to control for multiple comparisons. Effect sizes were calculated using partial eta squared (*η* ^2^p) for ANOVA effects and Cohen's *d* for pairwise contrasts. Results are reported as mean ± standard deviation (SD). To assess overall multivariate differences in interjoint coordination between groups, a one‐way MANOVA was conducted using coordination magnitude and variability as dependent variables. MANOVA was selected to reduce Type I error inflation and to account for correlations among coordination measures. In addition, statistical parametric mapping (SPM) was used to analyze continuous coupling‐angle waveforms across the normalized gait cycle (0%–100%). Within‐group pre–post differences and condition‐specific comparisons were performed using paired‐sample SPM{t} tests. These analyses were implemented in Python using the open‐source spm1d package. The SPM approach enabled the identification of significant temporal clusters along the time‐normalized gait cycle while controlling for the one‐dimensional and time‐dependent nature of waveform data. A significance threshold of *α* = 0.05 was adopted for all SPM analyses.

## 3 Results

Table 1 presents the demographic characteristics of the participants and compares these characteristics between the two groups.

**TABLE 1:** Demographic characteristics of the participants and comparison between the two groups.

	Groups		*p* value
	FFG (*n* = 12)	CG (*n* = 12)	
Sex (male/female)	5/7	5/7	
Age (y)	20.81 (2.7)	21.41 (2.9)	0.888
Height (m)	1.6 (0.07)	1.7 (0.07)	0.992
Weight (kg)	68.90 (10.23)	69.58 (12.22)	0.316
BMI	24.97 (2.42)	23.68 (2.45)	0.942

*Note:* Values are mean ± standard deviation. * Significance level *p* < 0.05.

Abbreviations: BMI, body mass index; CG, control group; FFG, experimental group; *n*, number of participants.

### 3.1 Spatiotemporal Parameters

Spatiotemporal outcomes are summarized in Table 2. Between‐group comparisons showed that step time during PNG was significantly longer in the CG than in the FFG (*p* < 0.05). Single support time was also longer in CG than FFG under NG, PNG, and PSH conditions (all *p* < 0.05). Use of antipronation insoles was associated with a significantly longer double support time in FFG relative to CG (*p* < 0.05). Under NG, stride length was significantly greater in CG than FFG (*p* < 0.05), whereas walking speed did not differ between groups (*p* > 0.05).

**TABLE 2:** Spatiotemporal parameters (mean ± SD) and between‐group comparison.

	FFG				CG			
	NG	PNG	SH	PSH	NG	PNG	SH	PSH
Step time (s)	0.55 ± 0.03	0.52 ± 0.06^a^	0.56 ± 0.04	0.55 ± 0.06	0.58 ± 0.05	0.58 ± 0.08	0.55 ± 0.04	0.54 ± 0.09
Single support (s)	0.42 ± 0.02^a^	0.40 ± 0.03^a^	0.41 ± 0.02	0.41 ± 0.09^a^	0.46 ± 0.04	0.45 ± 0.06	0.42 ± 0.02	0.45 ± 0.04
Double support (s)	0.24 ± 0.03	0.24 ± 0.07	0.27 ± 0.03^a^	0.26 ± 0.08	0.21 ± 0.04	0.21 ± 0.08	0.25 ± 0.03	0.25 ± 0.06
Stride length (m)	1.22 ± 0.04^a^	1.31 ± 0.09	1.34 ± 0.09	1.32 ± 0.09	1.29 ± 0.09	1.28 ± 0.07	1.38 ± 0.07	1.39 ± 0.07
Walking speed (m/s)	1.11 ± 0.09	1.24 ± 0.22	1.20 ± 0.11	1.19 ± 0.12	1.14 ± 0.16	1.16 ± 0.11	1.22 ± 0.10	1.24 ± 0.05

*Note:* Values are mean ± standard deviation.

Abbreviations: CG, control group; FFG, experimental group; NG, normal gait; PNG, Posttest normal gait; PSH, Posttest walking with shoes; SH, walking with shoes.

^a^ Indicates a significant difference between the two groups in the same condition.

### 3.2 Ankle–Hip Coupling

Between‐group comparisons revealed that long‐term use of antipronation insoles significantly reduced the coupling angle in the FFG compared to the CG during the LR (*p* = 0.009, *F* = 8.34, and *η* ^2^ *p* = 0.28) and S (*p* = 0.005, *F* = 9.90, and *η* ^2^ *p* = 0.32) phases under the NG condition. Additionally, after long‐term use, the coupling angle during the PO phase was significantly greater in the FFG than in the CG (*p* = 0.004, *F* = 10.29, and *η* ^2^ *p* = 0.33). The results also showed that immediate use of antipronation insoles led to a significant increase in the coupling angle during the PO phase (*p* = 0.02, *F* = 6.32, and *η* ^2^ *p* = 0.23). In the PSH condition, the coupling angle during the PO phase remained significantly higher in the FFG compared to the CG (*p* = 0.001, *F* = 13.46, and *η* ^2^ *p* = 0.39) (Table 3).

**TABLE 3:** Mean ± standard deviation of coupling angle in each gait subphase for the experimental (FFG) and control (CG) groups.

		FFG				CG			
		NG	PNG	SH	PSH	NG	PNG	SH	PSH
Ankle–hip	LR	278.17 ± 9.44 (PP)	264.41 ± 15.13^a^ (PP)	262.87 ± 17.44 (PP)	252.73 ± 15.42 (PP)	279.31 ± 9.13 (PP)	280.39 ± 7.87 (PP)	262.81 ± 11.76 (PP)	263.80 ± 10.82 (PP)
	MS	279.40 ± 10.46^a^ (PP)	275.70 ± 10.92 (PP)	282.12 ± 14.10 (PP)	280.15 ± 12.16 (PP)	269.90 ± 11.39 (PP)	268.23 ± 10.89 (PP)	274.18 ± 9.40 (PP)	274.74 ± 9.13 (PP)
	PO	109.16 ± 13.19 (PP)	121.47 ± 17.01^a^ (AP)	124.50 ± 15.42^a^ (AP)	124.62 ± 13.72^a^ (AP)	101.31 ± 13.02 (PP)	98.37 ± 12.90 (PP)	112.35 ± 6.29 (AP)	110.90 ± 5.99 (AP)
	S	139.51 ± 19.20 (AP)	124.86 ± 14.67^a^ (AP)	159.23 ± 21.55 (AP)	138.16 ± 37.91 (AP)	144.39 ± 15.03 (AP)	147.04 ± 16.28 (AP)	155.55 ± 41.20 (DP‐AP)	159.57 ± 8.42 (DP‐AP)
Ankle–knee	LR	152.26 ± 22.03 (AP)	155.10 ± 35.94 (DP‐AP)	173.91 ± 39.43 (DP)	169.23 ± 21.62 (DP)	152.92 ± 11.41 (AP)	151.33 ± 11.31 (AP)	183.09 ± 24.72 (DP)	179.89 ± 21.34 (DP)
	MS	147.04 ± 18.74 (AP)	151.36 ± 18.76 (AP)	140.59 ± 27.16 (AP)	147.91 ± 24.36 (AP)	155.48 ± 22.39 (AP)	155.33 ± 19.77 (AP)	147.07 ± 21.52 (AP)	145.87 ± 21.43 (AP)
	PO	209.28 ± 31.45 (DP‐IP)	214.68 ± 28.68 (DP‐IP)	228.68 ± 34.09 (IP)	236.09 ± 43.19 (IP)	188.75 ± 34.03 (DP)	180.31 ± 35.14 (DP)	214.16 ± 26.52 (IP)	208.74 ± 25.21 (IP)
	S	184.70 ± 13.71 (DP)	179.29 ± 16.17^a^ (DP)	172.32 ± 8.64 (DP)	168.53 ± 9.41^a^ (DP)	192.15 ± 13.64 (DP)	192.48 ± 15.28 (DP)	179.09 ± 9.26 (DP)	178.91 ± 8.13 (DP)
Knee–hip	LR	282.44 ± 13.69 (PP)	280.62 ± 36.94^a^ (PP)	284.60 ± 21.54 (PP)	266.94 ± 47.08 (PP)	290.36 ± 11.86 (PP)	289.25 ± 11.53 (PP)	288.17 ± 12.85 (PP)	284.51 ± 14.70 (PP)
	MS	258.76 ± 17.19 (PP)	249.81 ± 14.34 (PP)	259.53 ± 22.63 (PP)	256.39 ± 13.16 (PP)	246.04 ± 19.72 (PP‐IP)	243.90 ± 17.79 (PP‐IP)	250.78 ± 10.18 (PP)	249.47 ± 9.80 (PP)
	PO	37.86 ± 11.16 (IP)	49.93 ± 16.80 (IP)	41.05 ± 14.02 (IP)	42.15 ± 28.33 (IP)	40.21 ± 7.63 (IP)	42.40 ± 7.94 (IP)	35.18 ± 4.18 (IP)	35.61 ± 3.51 (IP)
	S	178.19 ± 20.52 (DP)	169.81 ± 18.48 (DP)	176.62 ± 15.73 (DP)	165.94 ± 10.04 (DP)	178.56 ± 17.21 (DP)	182.35 ± 12.70 (DP)	174.38 ± 15.56 (DP)	174.50 ± 16.17 (DP)

*Note:* Values are mean ± standard deviation.

Abbreviations: CG, control group; FFG, experimental group; LR, loading response; MS, mid‐stance; NG, normal gait; PNG, Posttest normal gait; PO, push‐off; PSH, Posttest walking with shoes; S, swing; SH, walking with shoes.

^a^ Indicates a significant difference between the two groups in the same condition.

**FIGURE 2:**
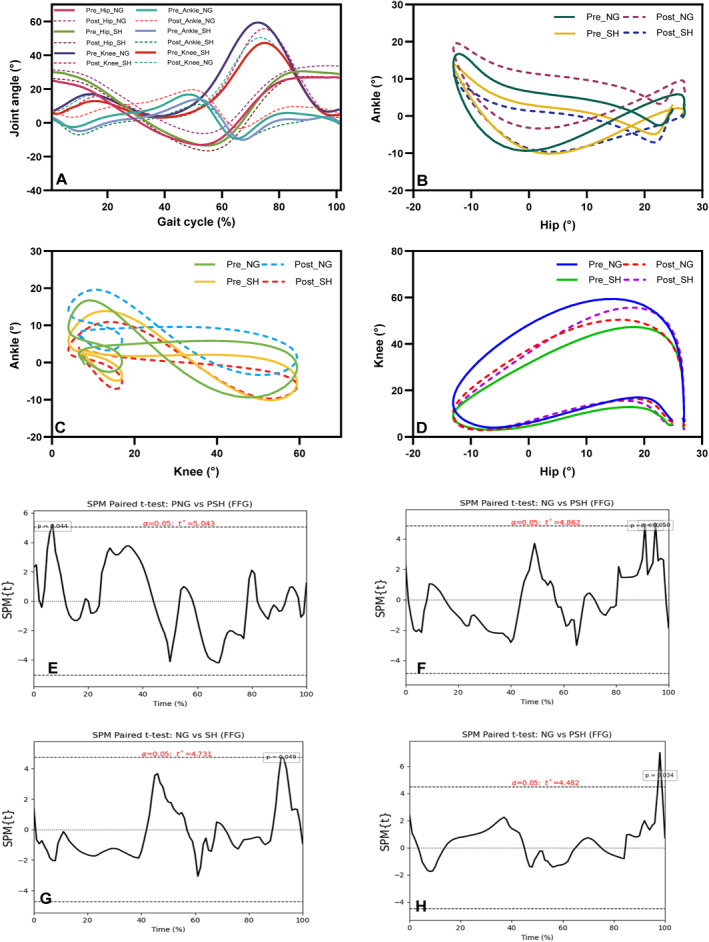
Joint kinematics and statistical parametric mapping (SPM) results for the flat‐foot group (FFG). (A) Sagittal plane joint angles of the ankle, knee, and hip during the gait cycle. (B–D) Angle–angle plots for ankle–hip, ankle–knee, and hip–knee joints under normal gait (NG), posttest normal gait (PNG), walking with shoes (SH), and posttest walking with shoes (PSH). (E) SPM results for ankle–hip coupling angles, (F–G) for ankle–knee coupling angles, and (H) for hip–knee coupling angles across gait conditions.

**FIGURE 3:**
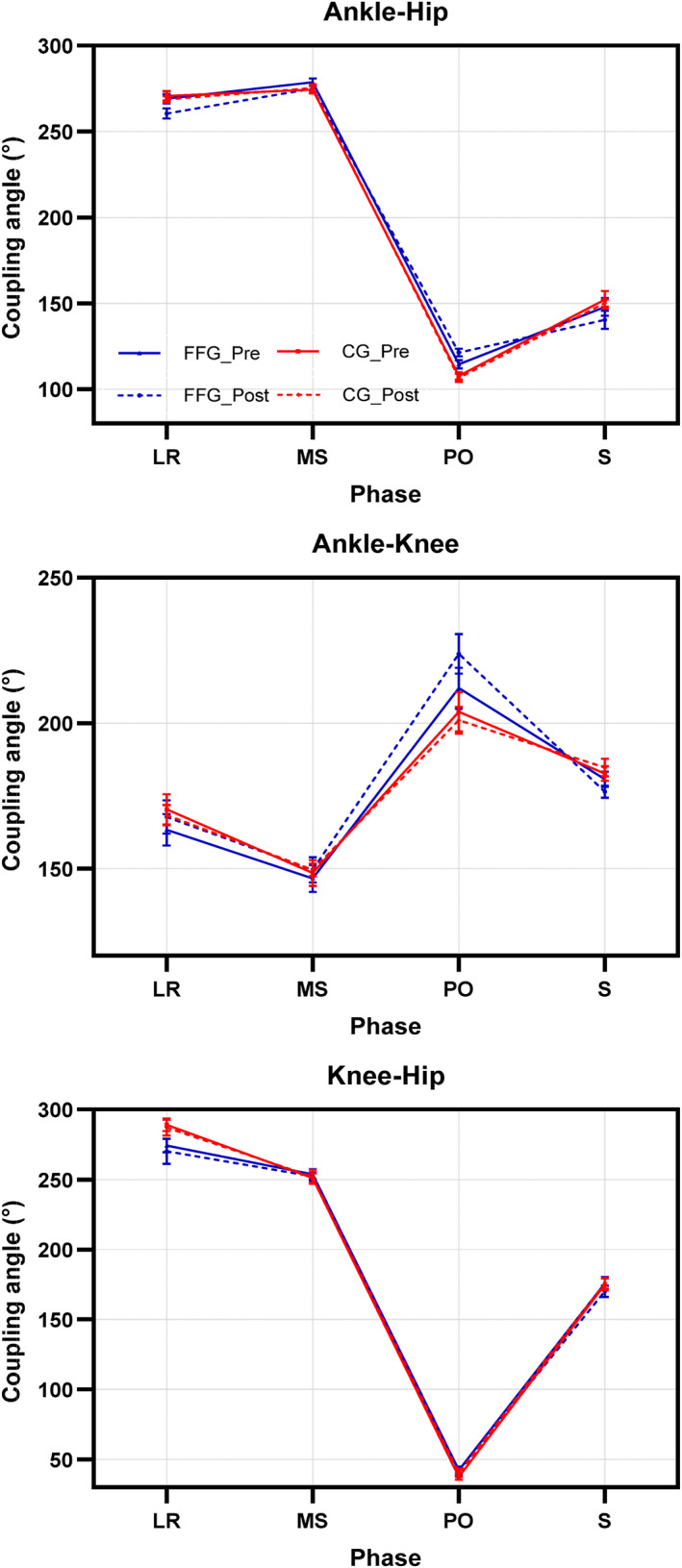
Interaction effect of group (FFG vs. CG), time (pretest vs. posttest), and gait phase on ankle–hip, ankle–knee, and hip–knee coupling angles (°) under normal gait (NG), posttest normal gait (PNG), walking with shoes (SH), and posttest walking with shoes (PSH). Gait phases: loading response (LR), mid‐stance (MS), push‐off (PO), and swing (S).

Factorial analysis showed that time had a significant effect on the coupling angle (*p* = 0.038, *F* = 4.91, and *η* ^2^ *p* = 0.19). Furthermore, the group × time interaction was also significant (*p* = 0.021, *F* = 6.25, and *η* ^2^ *p* = 0.23). Mean comparisons indicated that long‐term use of antipronation insoles contributed to a reduction in the coupling angle in the FFG. As shown in the SPM plot, this difference in the FFG between the with‐shoes and without‐shoes conditions was significant during the LR phase (Figure 2; *p* = 0.044). Additionally, the phase × group × time interaction was significant (*p* = 0.02, *F* = 4.16, and *η* ^2^ *p* = 0.40), indicating that, as illustrated in Figure 3, the FFG exhibited a higher coupling angle during the PO phase after long‐term use of antipronation insoles compared with the pretest condition.

**FIGURE 4:**
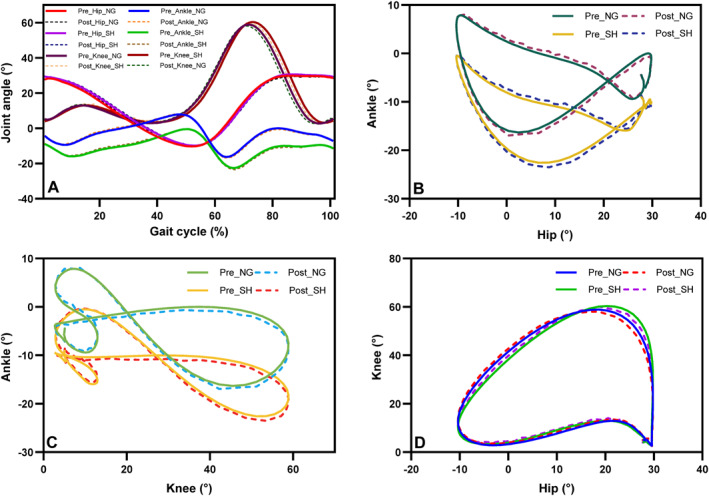
Joint kinematics for the control group (CG). (A) Sagittal plane joint angles of the ankle, knee, and hip during the gait cycle. (B–D) Angle–angle plots for ankle–hip, ankle–knee, and hip–knee joints under normal gait (NG), posttest normal gait (PNG), walking with shoes (SH), and posttest walking with shoes (PSH).

The insole (shoe) factor also had a significant effect, leading to an increase in the coupling angle (*p* = 0.005, *F* = 9.65, and *η* ^2^ *p* = 0.32). Factorial analysis further revealed a significant interaction between antipronation insole use and gait phase (*p* < 0.0001, *F* = 24.10, and *η* ^2^ *p* = 0.79; Figures 3 and 4). Mean comparisons showed that antipronation insoles had an immediate effect on all gait phases in both groups (*p* = 0.823, *F* = 0.30, and *η* ^2^ *p* = 0.05). However, the time × insole interaction was not significant (*p* = 0.507, *F* = 0.46, and *η* ^2^ *p* = 0.02). Mean comparisons, particularly in the FFG, indicated that long‐term use of antipronation insoles reduced the coupling angle in both NG and SH conditions. Nevertheless, antipronation insole and shoe use generally resulted in an overall increase in the coupling angle.

### 3.3 Ankle–Knee Coupling

Between‐group comparisons revealed that, following long‐term use of antipronation insoles, the FFG exhibited a lower coupling angle during the S phase while walking with shoes compared to the CG (*p* = 0.013, *F* = 7.34, and *η* ^2^ *p* = 0.26). No other significant between‐group differences were observed (Table 3). Factorial analysis indicated that the main effects of time (*p* = 0.565, *F* = 0.34, and *η* ^2^ *p* = 0.016) and the time × group interaction (*p* = 0.06, *F* = 3.94, and *η* ^2^ *p* = 0.16) on the coupling angle were not significant. However, the phase × time interaction was significant (*p* = 0.026, *F* = 4.88, and *η* ^2^ *p* = 0.38). SPM analysis revealed that long‐term use of anti‐pronation insoles led to an approximately 11° increase in the coupling angle during the S phase (Figure 2, *p* < 0.05). Additionally, the main effect of the insole factor resulted in a significant increase in the coupling angle (*p* < 0.001, *F* = 27.31, and *η* ^2^ *p* = 0.56). A significant insole × phase interaction revealed that immediate use of anti‐pronation insoles increased the coupling angle by approximately 24° in the LR phase and 11° in the S phase (*p* < 0.001, *F* = 16.29, and *η* ^2^ *p* = 0.72).

### 3.4 Knee–Hip Coupling

The results indicated that the between‐group difference in knee–hip coupling was not significant, except in the LR phase under the PNG condition, where it was significantly lower in the FFG compared to the CG (*p* = 0.046, *F* = 4.52, and *η* ^2^ *p* = 0.18) (Table 3). Factorial analysis showed that the main effects of time (*p* = 0.223, *F* = 1.57, and *η* ^2^ *p* = 0.07) and the time × group interaction (*p* = 0.435, *F* = 0.63, and *η* ^2^ *p* = 0.03) on the coupling angle were not significant. Only the main effect of shoes resulted in a significant decrease in the knee–hip coupling angle (*p* = 0.003, *F* = 11.34, and *η* ^2^ *p* = 0.35). The SPM analysis demonstrated that long‐term use of antipronation insoles led to an increase in the coupling angle during the PO phase in the FFG (Figure 2, *p* < 0.05).

## 4 Discussion

To gain insight into interjoint coordination, it is essential to examine both the immediate and long‐term effects of using an antipronation insole on sagittal plane coordination during gait in individuals with flat feet. This study aimed to explore these effects, providing a deeper understanding of how antipronation insoles use influences movement patterns in this population.

### 4.1 Ankle–Hip Coordination

This study demonstrates that long‐term use of antipronation insoles significantly alters interjoint coordination during walking in individuals with flat feet as reflected in phase‐dependent changes in coupling angles and spatiotemporal variables. The reduction in coupling angles during the LR and S, along with the increase during PO, suggests a reorganization of lower limb kinematics, which warrants further biomechanical investigation. These phase‐dependent changes align with previous research showing that insoles modulate intersegmental coordination through mechanical and neuromuscular mechanisms [29]. The reduction in coupling angles during the LR and S phases in the FFG postintervention is likely due to improved control of excessive pronation, which otherwise disrupts proximal‐to‐distal energy transfer at the beginning of stance. This finding supports Chang et al. (2008), showing antiphase coordination after prolonged insole use [35]. Increased coupling angles during PO suggest enhanced propulsion, likely due to improved windlass mechanism function [38]. The group‐phase‐time interaction highlights the complexity of insole effects, with FFG showing phase‐dependent adaptations similar to athletes [39]. Reduced coupling angles in LR and S, alongside increased PO angles, resemble strategies used by soccer players to optimize joint stiffness [40]. This adaptation may help conserve energy in individuals with chronic foot abnormalities.

The spatiotemporal adaptations observed after 6 weeks of insole use should be interpreted cautiously. Although the flatfoot group demonstrated increased double‐support time and slightly longer stride length, these changes do not necessarily reflect improved dynamic stability. In the gait adaptation literature, prolonged double‐support time is frequently described as a conservative or compensatory strategy aimed at increasing ground‐contact duration to enhance perceived steadiness rather than representing intrinsic improvements in neuromuscular control [41, 42]. Such adaptations are often observed in individuals with altered foot mechanics or reduced distal joint stiffness. Therefore, the present results may indicate a cautious gait strategy adopted during adaptation to modified foot alignment rather than a direct enhancement in stability. Confirming true improvements in dynamic stability would require specific stability metrics—such as center of mass displacement, trunk acceleration, or margin of stability analyses—which were beyond the scope of this study. However, the persistent difference in single support time highlights the need for longer adaptation periods to achieve complete gait normalization. These findings align with Peng et al. (2020), who reported progressive dual‐task gait improvements after 1 week of insole use, suggesting that extended intervention durations may yield greater benefits [43].

Clinically, the phase‐dependent effects of antipronation insoles emphasize the importance of task‐specific rehabilitation strategies. The significant interaction between antipronation insoles use and gait phase suggests that insoles are most effective during the LR and PO phases, where flat feet exhibit the greatest kinematic deviations [44]. This phase‐specific efficacy should guide the integration of insoles with physical therapy to optimize rehabilitation outcomes.

### 4.2 Ankle–Knee Coordination

The significant increase in ankle–knee coupling angles during PO and S phases after prolonged antipronation insoles use suggests enhanced joint coordination. The 11‐degree rise in coupling angles during these phases aligns with previous findings indicating that arch‐support insoles improve sagittal plane synchronization by reducing excessive tibial internal rotation—a common compensatory pattern in flat‐footed gait [45]. This effect likely results from the insoles' ability to limit subtalar pronation, mitigating abnormal tibiofemoral kinematics. The 24‐degree increase in coupling angles during LR with initial insole application suggests rapid neuromotor adaptation, possibly mediated by proprioceptive feedback. However, the lack of significant time × group interactions implies that prolonged use induces gradual neuromuscular recalibration rather than sudden kinematic shifts, consistent with studies on neurophysiological adaptations to insole use [46]. These phase‐dependent changes highlight the stance phase as critical for antipronation insoles efficacy. During LR, insoles may enhance medial longitudinal arch energy storage, reducing knee stabilizer demands [47]. In PO, improvements likely stem from optimized force transfer via restored arch mechanics as supported by instrumented insole studies showing increased propulsion efficiency [48]. These findings align with vector coding analyses demonstrating altered intersegmental coordination in flatfoot populations during mid‐stance [45].

### 4.3 Knee–Hip Coordination

Knee–hip coupling did not show significant time‐dependent changes following long‐term use of antipronation insoles, indicating that proximal coordination remained relatively stable. The only significant effect was related to the footwear condition, which produced a modest reduction in coupling magnitude irrespective of group or intervention. Because this reduction was not linked to the insole treatment and no meaningful time × group effects were observed, it should be interpreted as a footwear‐related mechanical influence rather than a specific consequence of antipronation insole use. The persistence of hip–knee coordination aligns with studies in pediatric populations using arch supports, where proximal adjustments lag behind distal improvements [29]. This hierarchical adaptation may reflect the central nervous system's prioritization of ankle–foot stability over hip adjustments during gait rehabilitation [47]. FFG participants exhibited shorter step times and single‐support durations compared CG, along with increased double‐support time during antipronation insoles use. These spatiotemporal modifications indicate a shift toward stability over propulsion, a common pattern in individuals with altered foot mechanics [45]. Despite reduced step length, comparable walking speeds across groups suggest cadence adjustments to maintain velocity, potentially increasing metabolic cost. This trade‐off between stability and efficiency should be considered when prescribing insoles for functional activities.

One limitation of the present study is that subtalar joint kinematics were not directly measured. Given the key role of subtalar pronation in mediating distal‐to‐proximal coupling mechanisms, future research should incorporate multi‐segment foot models—such as the Oxford Foot Model—to quantify subtalar motion and better elucidate its contribution to interjoint coordination patterns in individuals with flatfoot.

## 5 Conclusion

This study showed that long‐term use of antipronation insoles produced phase‐specific changes in distal interjoint coordination in individuals with flat feet, particularly at the ankle–hip joint, whereas proximal coordination patterns remained largely unchanged. These findings indicate that insoles can influence distal joint interactions during gait; however, the spatiotemporal changes observed—such as increased double‐support time and slight modifications in stride parameters—should be interpreted as cautious or conservative gait adjustments rather than evidence of improved dynamic stability. Because the study did not include direct measures of gait stability (e.g., center‐of‐mass displacement, trunk accelerometry, or margin‐of‐stability indices), no definitive conclusions can be drawn regarding stability improvements. Additionally, the relatively small sample size and the absence of EMG or plantar pressure data limit the ability to fully characterize the neuromuscular and foot–ground mechanisms underlying the observed coordination changes. Future studies incorporating larger samples and multimodal biomechanical assessments are needed to more comprehensively evaluate the long‐term effects of antipronation insoles on gait dynamics and to determine whether combining orthotic interventions with targeted rehabilitation exercises yields greater proximal adaptations.

## Author Contributions

**Negin Soltani:** conceptualization, methodology, investigation, data curation, formal analysis, writing – review and editing. **Mahdi Majlesi:** conceptualization, methodology, formal analysis, writing – original draft, writing – review and editing, supervision. **Ali Fatahi:** investigation, data curation, formal analysis, writing – review and editing.

## Funding

The authors have nothing to report

## Ethics Statement

This study was approved by the Ethics Committee of Islamic Azad University, Hamedan Branch (Approval Code: IR.IAU.H.REC.1402.130 and Approval Date: 19 November 2023) and conducted in accordance with the ethical standards of the institutional research committee and with the 1964 Declaration of Helsinki and its later amendments. Written informed consent was obtained from all participants prior to their inclusion, and their confidentiality and right to withdraw from the study at any stage were fully respected.

## Conflicts of Interest

The authors declare no conflicts of interest.

## Data Availability

The data that support the findings of this study are available from the corresponding author upon reasonable request.
